# *Helicteresbinhthuanensis* V.S.Dang (Malvaceae, Helicteroideae), a new species from southern Vietnam

**DOI:** 10.3897/phytokeys.166.57647

**Published:** 2020-10-29

**Authors:** Van-Son Dang, Minh-Quan Dang, Nghia-Son Hoang

**Affiliations:** 1 Institute of Tropical Biology, Vietnam Academy of Science and Technology, 85 Tran Quoc Toan Street, District 3, Ho Chi Minh City, Vietnam Vietnam Academy of Science and Technology Ho Chi Minh Vietnam; 2 School of Education, Can Tho University, 3/2 Street, Ninh Kieu District, Can Tho City, Vietnam Graduate University of Science and Technology Ha Noi Vietnam; 3 Graduate University of Science and Technology, Vietnam Academy of Science and Technology, 18 Hoang Quoc Viet Street, Cau Giay District, Ha Noi City, Vietnawm Can Tho University Can Tho Vietnam

**Keywords:** *Helicteres*, Helicteroideae, Malvaceae, Vietnam

## Abstract

*Helicteresbinhthuanensis* V.S.Dang, **sp. nov.** from Ham Thuan Bac District, Binh Thuan Province, Vietnam is described and illustrated. It is morphologically similar to *H.angustifolia*, which is a common species in mainland southeast Asia, and *H.sphaerotheca*, which is endemic to the Northern Territory, Australia, but differs from both by several salient characters such as leaf and calyx size, androgynophore length, petal color, and fruit shape. Photographs, a vernacular name, a preliminary conservation assessment, and a table of morphological characters comparing this new species to two closely related species also are provided.

## Introduction

*Helicteres* L. comprises about 60 species in the tropics of Asia and America (Cowie 2011). The genus was included in a broadly-defined Sterculiaceae by Cronquist (1981), but more recently is included in Malvaceae*sensu lato* based on morphological and molecular evidence (Mabberley 2008; APG IV 2016). The genus is characterized by the stamens and pistil forming an androgynophore, sepals united, petals unguiculate, capsules cylindrical, fusiform or ovoid, spiral or rarely straight, pubescent, and seeds wingless due to parenchymal expansion (Cristóbal 2001; Chantaranothai and Poompo 2019). Currently, nine species and one variety of the genus have been recorded in Vietnam: *H.angustifolia* L., H.angustifoliavar.glaucoides Pierre, *H.daknongensis* V.S.Dang & D.T.Bui, *H.elongata* Wall. ex Mast., *H.hirsuta* Lour., *H.isora* L., *H.lanata* (Teijsm. & Binn.) Kurz, *H.lanceolata* DC., *H.poilanei* Tardieu, and *H.viscida* Blume (Hoang et al. 2020).

**Figure 1.**
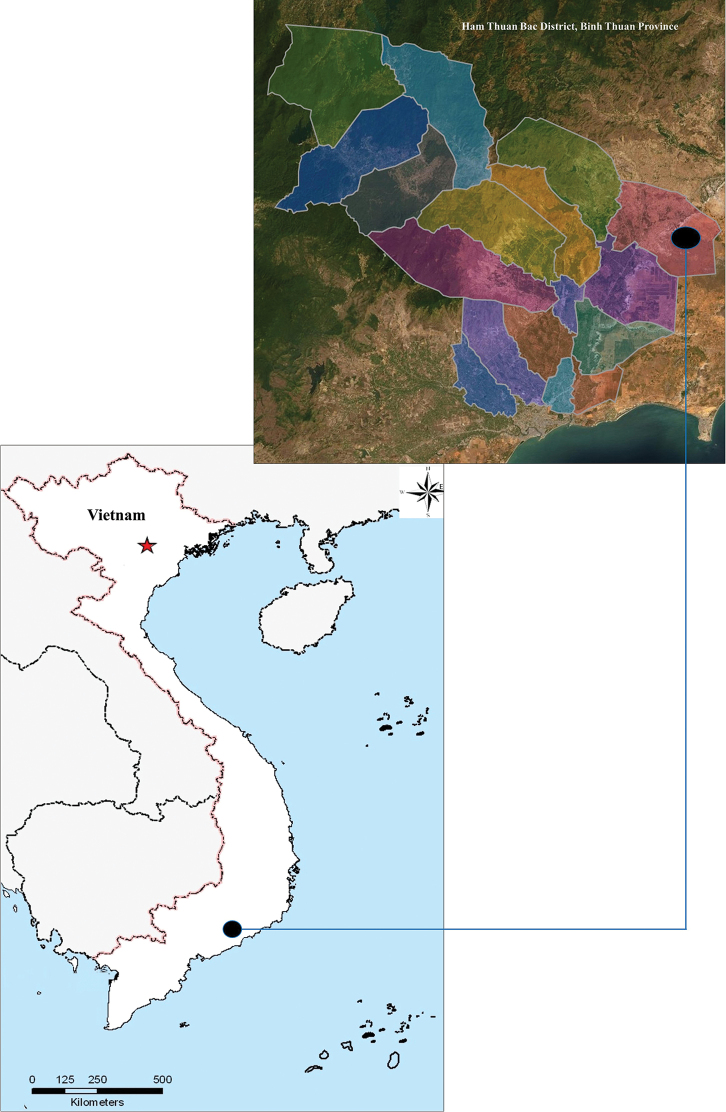
Type locality of *Helicteresbinhthuanensis*.

During fieldwork in July 2019, we collected several specimens of *Helicteres* in Ham Thuan Bac District, Binh Thuan Province, southern Vietnam (Figure 1). After morphological study and reviewing the relevant literature from Vietnam and neighboring countries (Tardieu-Blot 1945; Pham 1999; Cristóbal 2001; Phengklai 2001; Nguyen 2003; Tang et al. 2007; Cowie 2011), as well as comparing our material with specimens in several Vietnamese herbaria (HN, VNM, and VNMN) and images available via JSTOR Global Plants, K, and P (all acronyms follow Thiers 2020, continuously updated), we confirm that this material is distinct from previously known taxa. Here, we describe it as a new species, *H.binhthuanensis* V.S.Dang.

## Materials and methods

Our description of the new species is based on observations from living and dried specimens; measurements were made using a ruler accurate to 0.5 mm. Voucher specimens were deposited in HN, VNM, and VNMN. The photographs were taken with a Canon 1000D camera fitted with an EF 100 mm f/2.8 Macro USM lens. The conservation status of the new species was assessed according to the International Union for Conservation of Nature (IUCN 2019).

## Taxonomic treatment

### Helicteres binhthuanensis

Taxon classificationPlantae

V.S.Dang sp. nov.

DB96B855-5B8F-56AB-A77D-97F20F779370

urn:lsid:ipni.org:names:77212606-1

Figures 2 , 3

#### Diagnosis.

Similar to *Helicteresangustifolia* L. (Figure 4) in its shrubby habit (to 2 m tall) and axillary inflorescences but distinguished by the size of its leaf blades (10–13 × 0.8–1.2 cm vs. 3–15 × 1–4 cm), larger petals (12–16 mm long vs. 5–10 mm long), longer androgynophores (10–15 mm long vs. 3–7.5 mm long), and stamen number (10 vs. 15). Also similar to *H.sphaerotheca* Cowie, but differs from the latter by having larger leaf blades (10–13 × 0.8–1.2 cm vs. 1.5–3.5 × 0.2–0.7 cm), longer stipules (3–4 mm long vs. 1–2 mm long) and bracts (1–2 mm long vs. 1 mm long), larger calyx (tube 6–8 mm long, lobes 4–5 mm long vs. tube 3.5–4.5 mm long, lobes ca. 0.5 mm long) and petals (12–16 mm long vs. 7–9.5 mm long), and fruit shape (ovoid to ellipsoid vs. globular).

**Figure 2.**
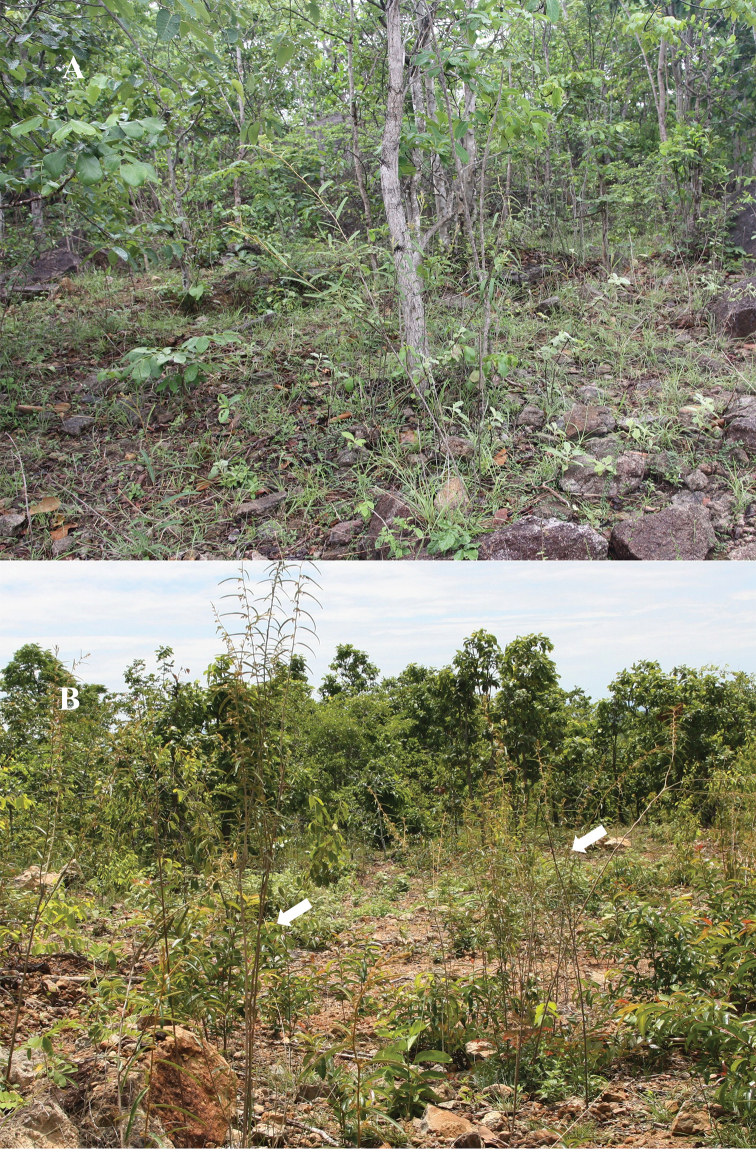
Habitat and habit of *Helicteresbinhthuanensis***A** habitat **B** habit. Photos by Van-Son Dang.

**Figure 3.**
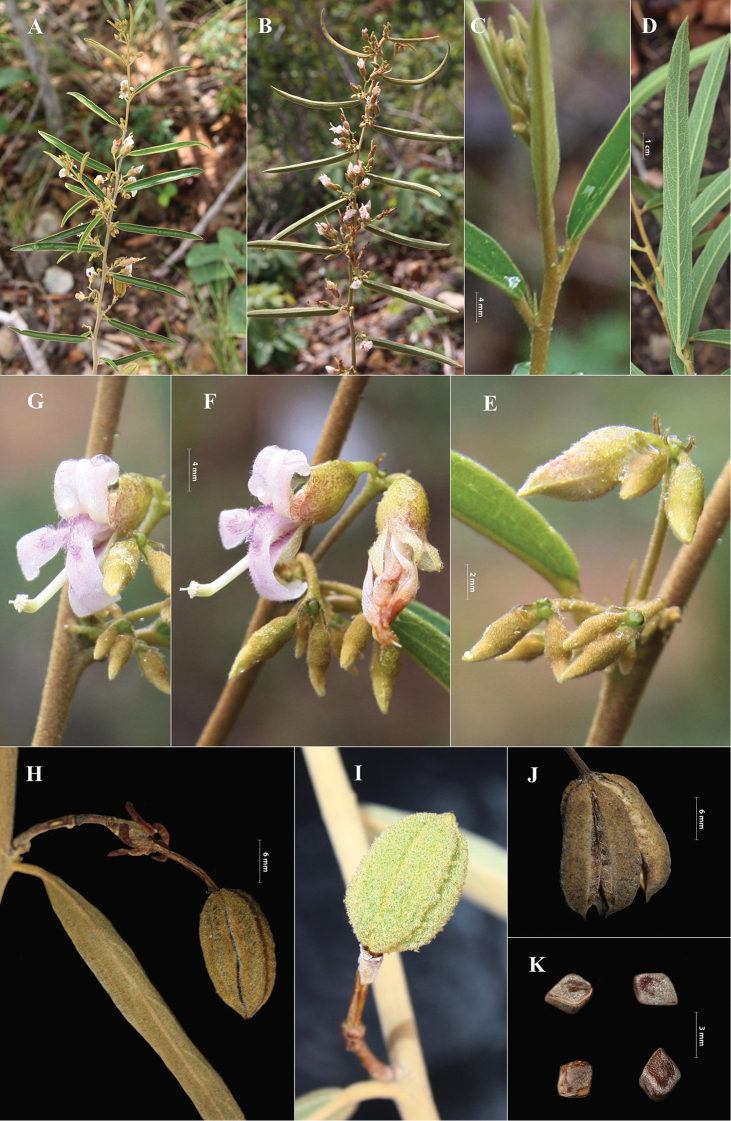
*Helicteresbinhthuanensis***A, B** flowering and fruiting branches **C** close-up of young leaves and stipules **D** abaxial leaf surface **E–G** close-up of axillary inflorescence and flowers **H–J** close-up of fruits **K** Seeds. Photos by Van-Son Dang.

**Figure 4.**
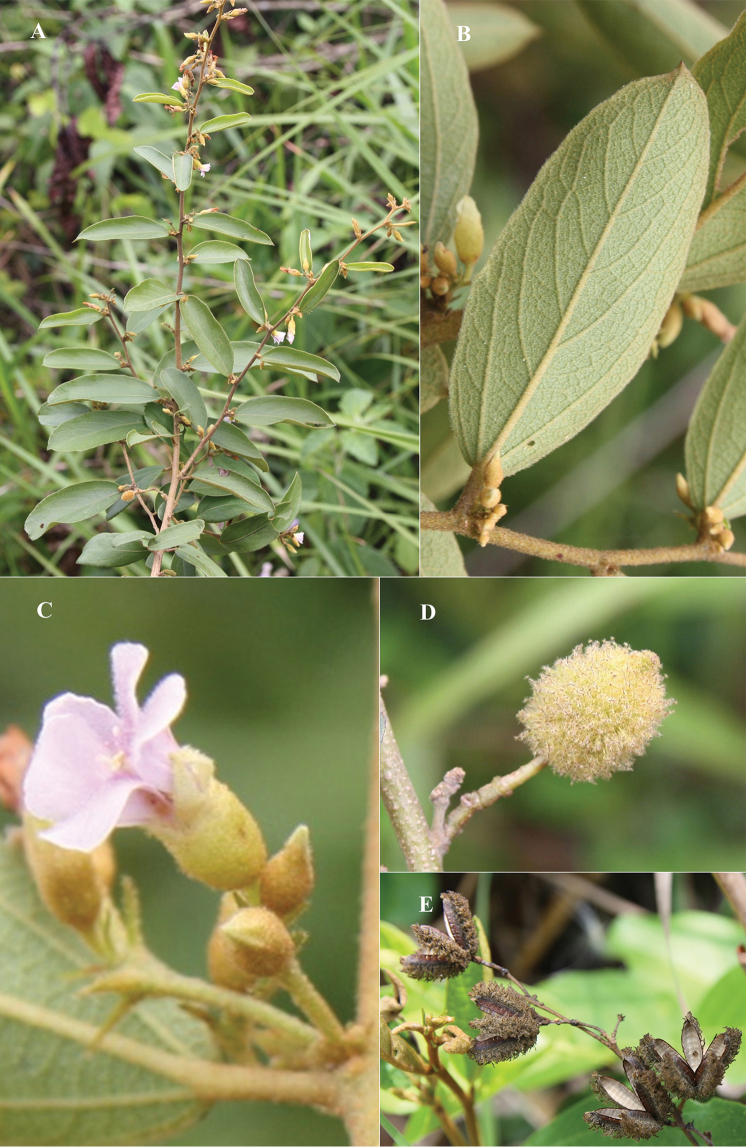
*Helicteresangustifolia***A** flowering branches **B** abaxial leaf surfaces **C** close-up of axillary inflorescence and flowers **D, E** close-up of fruits. Photos by Van-Son Dang.

#### Type.

Vietnam. Binh Thuan Province, Ham Thuan Bac District, 40 km north of Phan Thiet City, in secondary forests, 11°07’02.30”N, 108°14’92.07”E, 80 m alt., 20 July 2019, *Van-Son Dang & Nghia-Son Hoang, Dang 386* (holotype: VNM; isotypes: HN, VNM, VNMN).

#### Description.

Shrubs, 1–2 m tall; branches slender, 2–3 mm in diam., densely yellow-brown stellate puberulent. Leaves alternate, blades narrowly lanceolate to narrowly oblanceolate, 10–13 × 0.8–1.2 cm, coriaceous, yellowish brown when dry, adaxially sparsely puberulent, abaxially densely yellow-brown puberulent, apex acute or acuminate, base rounded, symmetric, margin entire; midrib flat or slightly depressed adaxially, distinct and prominent abaxially; secondary veins 3–7 pairs, obscure adaxially, prominent abaxially; petioles 5–8 mm long, densely yellow-brown puberulent. Stipules persistent, 3–4 mm long, filiform or linear, densely pubescent. Inflorescences axillary or terminal, cymose, 8–14 mm long, 2–5-flowered; bracts 1–2 mm long; pedicels 1–5 mm long. Flowers with short pedicel; calyx tubular to campanulate, 10–13 mm long, whitish green, densely villous to hirsute, calyx tube 6–8 mm long, calyx lobes 5, unequal, lanceolate to triangular, 4–5 mm long, tips acute; petals 5, unequal in length, 12–16 mm long, whitish pink or purplish, darker at base of limb, reflexed at anthesis, limb cuneate, densely hirsute, callused near base, apex truncate, lower 3 petals slightly longer than upper pair, claw with 2 or 1 auriculate appendices, upper pair with 2 prominent appendages on the claw; androgynophore 10–15 mm long, straight, villous at base; stamens 10, shortly connate at base; staminodes 5, broadly lanceolate; filaments coalescent, surrounding ovary; anthers transverse, oblong; ovary ovoid or globose, 5-locular, densely villous; style 1.3–1.5 mm long; stigma 5-toothed, terete. Fruit a capsule, ovoid to ellipsoid, 1.5–2 × 0.8–1 cm, with 5 longitudinal lobes, densely villous, apex short-beaked, black when mature; seeds many, small, 2.5–4 × 1.5–2 mm, irregularly rugose, dark brown, angled when dry.

#### Other specimen examined.

Vietnam. Binh Thuan Province, Ham Thuan Bac District, 40 km north of Phan Thiet City, in secondary forests, 11°07’02.33”N, 108°14’92.27”E, 86 m elevation, 21 July 2019, *Van-Son Dang & Nghia-Son Hoang, Dang 383* (VNM).

#### Phenology.

Flowering and fruiting specimens were collected in July.

#### Distribution and habitat.

*Helicteresbinhthuanensis* is known only from Ham Thuan Bac District, Binh Thuan Province, southern Vietnam. It grows along roadsides and edges of secondary forests at ca. 80–95 m elevation.

#### Etymology.

The species epithet is derived from the name of the province (Binh Thuan) where the species was discovered.

#### Vernacular names.

Tổ kén bình thuận, An xoa bình thuận.

#### Preliminary conservation assessment.

*Helicteresbinhthuanensis* was collected from a small population in a habitat that was logged and disturbed. Therefore, we suggest its placement in the Data Deficient (DD) category based on the IUCN Red List Categories (IUCN 2019).

#### Notes.

Morphologically, *Helicteresbinhthuanensis* is most similar to *H.angustifolia*, which is a common species in mainland southeast Asia, and *H.sphaerotheca*, which is endemic to the Northern Territory of Australia, between the Mary River and the South Alligator River. It differs from those species in several characters that are summarised in Table 1.

**Table 1.** Comparison of *Helicteresbinhthuanensis* with its morphologically closest allies (modified from Pham 1999; Phengklai 2001; Tang et al. 2007; Cowie 2011).

Character	*H.binhthuanensis*	*H.angustifolia*	*H.sphaerotheca*
Branch indumentum	densely yellow-brown stellate puberulent	gray greenish puberulent	sparsely stellate hairy, hairs sessile
Petiole length	5–8 mm long	3–15 mm long	0.5–1 mm long
Leaf blade size	10–13 × 0.8–1.2 cm	3–15 × 1–4 cm	1.5–3.5 × 0.2–0.7 cm
Stipule length	3–4 mm long	3.5–6 mm long	1–2 mm long
Bract length	1–2 mm long	1.5–2 mm long	ca. 1 mm long
Calyx tube length	6–8 mm long	ca. 6 mm long	3.5–4.5 mm long
Calyx lobe length	4–5 mm long	3–4 mm long	ca. 0.5 mm long
Petal color at anthesis	whitish pink or purplish	bluish or pink	mauve-pink
Petal length	12–16 mm long	5–10 mm long	7–9.5 mm long
Androgynophore length	10–15 mm long	3–7.5 mm long	4.5–5 mm long
Number of stamens	10	15	10
Number of staminodes	5	O	5
Fruit shape	ovoid to ellipsoid	ovate to oblong	globular
Seed length	2.5–4 mm long	2–3.5 mm long	ca. 2.5 mm long

## Supplementary Material

XML Treatment for Helicteres binhthuanensis
